# Metabolic preconditioning in CD4^+^ T cells restores inducible immune tolerance in lupus-prone mice

**DOI:** 10.1172/jci.insight.143245

**Published:** 2021-10-08

**Authors:** Christopher S. Wilson, Blair T. Stocks, Emilee M. Hoopes, Jillian P. Rhoads, Kelsey L. McNew, Amy S. Major, Daniel J. Moore

**Affiliations:** 1 Ian Burr Division of Endocrinology and Diabetes, Department of Pediatrics;; 2 Department of Pathology, Microbiology, and Immunology; and; 3 Division of Cardiovascular Medicine, Department of Medicine, Vanderbilt University Medical Center, Nashville, Tennessee, USA.

**Keywords:** Autoimmunity, Transplantation, Autoimmune diseases, Lupus, Tolerance

## Abstract

Autoimmune disease has presented an insurmountable barrier to restoration of durable immune tolerance. Previous studies indicate that chronic therapy with metabolic inhibitors can reduce autoimmune inflammation, but it remains unknown whether acute metabolic modulation enables permanent immune tolerance to be established. In an animal model of lupus, we determined that targeting glucose metabolism with 2-deoxyglucose (2DG) and mitochondrial metabolism with metformin enables endogenous immune tolerance mechanisms to respond to tolerance induction. A 2-week course of 2DG and metformin, when combined with tolerance-inducing therapy anti-CD45RB, prevented renal deposition of autoantibodies for 6 months after initial treatment and restored tolerance induction to allografts in lupus-prone mice. The restoration of durable immune tolerance was linked to changes in T cell surface glycosylation patterns, illustrating a role for glycoregulation in immune tolerance. These findings indicate that metabolic therapy may be applied as a powerful preconditioning to reinvigorate tolerance mechanisms in autoimmune and transplant settings that resist current immune therapies.

## Introduction

Systemic lupus erythematosus (SLE) is an autoimmune disease that is characterized by inappropriate B and T cell collaboration leading to T cell activation and autoantibody production (1). In SLE, pathogenic autoantibodies directed against nuclear antigens collect in the kidney, occlude nephrons, and activate complement to cause nephritis (2, 3). Patients can develop severe kidney damage and may require kidney transplants, which are subject to both recurrent autoimmunity as well as allogeneic rejection, placing patients with SLE at a higher risk of graft dysfunction and loss (4). Studies have indicated that autoreactive effector T cells in SLE partially resist immune regulation, which poses a barrier to immune therapy (5).

In healthy individuals in the absence of immune insult or infection, the majority of T cells remain in an unreactive, naive state. This state is marked by relatively reduced metabolic requirements, fulfilled by low levels of mitochondrially driven oxidative phosphorylation (OXPHOS) to produce ATP (6). Once CD4^+^ T cells become activated, they undergo a metabolic switch to increase OXPHOS and glycolysis (6, 7). These metabolic processes prepare T cells to carry out effector functions by providing precursors for synthesis of macromolecules important for cell function and by regulating homing receptors that retain these CD4^+^ T cells in secondary lymphoid organs. In SLE, there is a seemingly spontaneous increase in activated CD4^+^ T cells. CD4^+^ T cells from murine models and humans with SLE demonstrate exaggerated mitochondrial OXPHOS and glycolysis compared with healthy controls (8, 9). It is unclear whether enhanced CD4^+^ T cell metabolism leads to spontaneous activation or whether heightened metabolism represents the activated state of CD4^+^ T cells actuated via some other mechanism. Nonetheless, enhanced metabolism is functionally related to the pathogenesis caused by CD4^+^ T cells in SLE.

Targeting glycolysis and OXPHOS via 2-deoxyglucose (2DG) and metformin normalized CD4^+^ T cell metabolism and reduced pathogenic CD4^+^ T cells in SLE mouse models (9). Continuous inhibition of glucose metabolism and OXPHOS prevents the production of autoantibodies and the onset of lupus-like disease in a robust animal model of SLE, *B6.Sle1.Sle2.Sle3* (referred to as SLE123 mice for the rest of this manuscript) (9–13). Treatment prompted changes in immunologic phenotypes and ultimately disease pathology; however, this treatment needed to be provided continuously to prevent reemergence of autoreactive processes.

Regulation of cellular metabolism is closely linked to intracellular signaling cascades that are controlled downstream of the T cell receptor (7, 14, 15). Enhanced AKT/mTOR signaling has been described in CD4^+^ T cells from humans and mice with SLE (16–18). This observation demonstrates integrated regulation of signaling and metabolism within CD4^+^ T cells, which likely drives autoimmune effector function. CD45 is a cell membrane phosphatase that plays a prominent role in regulating cell signaling proximal to the antigen receptor in both T cells and B cells. CD45 is functionally aberrant in many forms of autoimmunity, including SLE, leading to abnormal cellular development and function (19–21). Targeting the CD45RB isoform of CD45 with a monoclonal antibody induces tolerance to allografted organs in nonautoimmune prone mice but fails in SLE prone mice, suggesting that these abnormal signals and their downstream effects are key checkpoints in tolerance induction (22–27).

The failure of SLE mice to establish a tolerance-inducing response to treatment may relate to their abnormal metabolic processes, which also have the capacity to modulate signaling by altering ATP availability, calcium flux, reactive oxygen species, and protein function. As such, we interrogated whether altered CD4^+^ T cell metabolism in the SLE background inhibits tolerogenic signaling in response to therapy. We determined that tolerance induction by anti-CD45RB robustly alters metabolic genes in tolerance-permissive B6 mice that led to changes in glucose uptake and mitochondrial function. These changes did not occur in resistant SLE123 mice. Treatment of SLE123 with 2DG and metformin for 2 weeks along with anti-CD45RB enhanced the efficacy of anti-CD45RB and resulted in increased tolerance to allografted islets. Additionally, this 2-week treatment course reduced anti-dsDNA titers and antibody deposition for over 6 months when delivered as a short course to 9-week-old SLE123 mice. Overall, this work demonstrates abnormal metabolism is a potent barrier to immune tolerance that can be modified to allow successful tolerance induction.

## Results

### Anti-CD45RB promotes regulation of the B and T lymphocyte compartment that is resisted in the SLE123 mouse.

**Figure 1:**
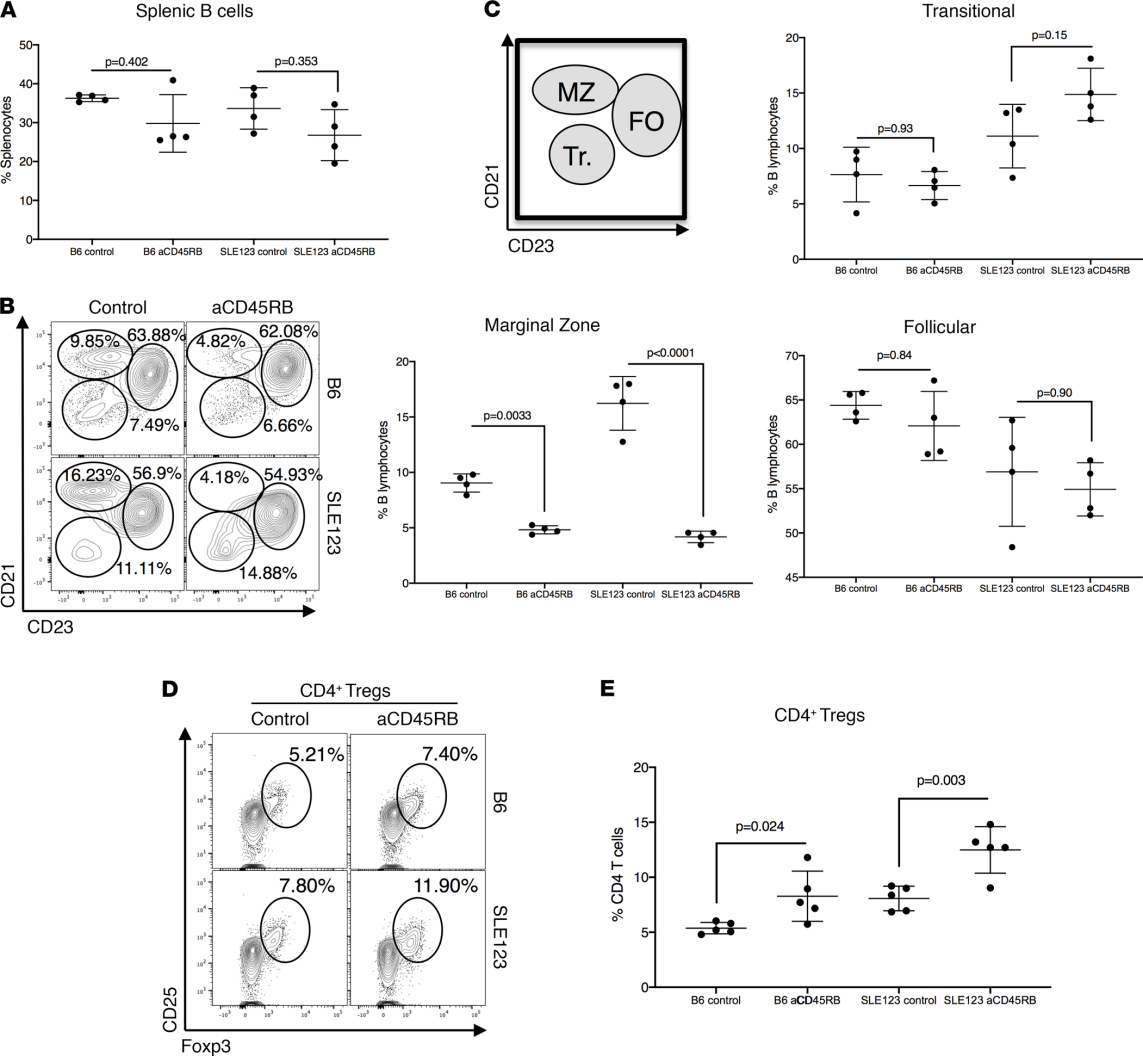
Tolerance-inducing anti-CD45RB mobilizes regulation in both the B and T lymphocyte compartments in B6 and SLE123 mice. (**A**) A 7-day course of aCD45RB was administered to B6 and SLE123 mice. Flow cytometry analysis of B6 and SLE123 mice (*n* = 4 per group, 9- to 10-week-old females) demonstrated a slight reduction in splenic B cells in both strains. (**B**) Subset analysis of the B cell compartment in these mice revealed a reduction in the marginal zone (MZ) while the Follicular (FO) and Transitional (Tr.) did not show substantial changes. (**C**) A cartoon diagram demonstrates the flow cytometry subsetting strategy, with quantification at right. Analyzed using a 1-way ANOVA followed by Tukey’s multiple comparison test. (**D** and **E**) Flow cytometry analysis showed B6 mice experienced an expansion of CD4^+^CD25^+^Foxp3^+^ Tregs when treated with aCD4RB (top panel). The SLE123 mice experienced a similar increase in the cells (bottom panel). This increase is quantified in **E**. Analyzed using a 1-way ANOVA followed by Tukey’s multiple comparison test. Analysis was carried out in 9- to 12-week-old female mice, *n* = 5 per group.****

We previously established that SLE123 mice resist transplant tolerance induced by a monoclonal antibody (MB23G2) that targets cell surface phosphatase CD45RB (27). Tolerance to transplantation was resisted by SLE123 mice even when the transplanted organ was not the target of recurrent autoimmunity as has also been observed in other autoimmune models (23, 28–30). To rule out the contribution of cellular abnormalities and activation that emerge later in the life of SLE123 mice, we assessed the response of 9- to 12-week-old mice in this study prior to the detection of chronic immune cell activation. Regulation of graft rejection by anti-CD45RB requires mobilization of both the B cell compartment and Tregs (22, 24, 31). A 7-day course of anti-CD45RB treatment indicated a trend toward reduction in B cells in both strains but did not reach statistical significance (Figure 1A). An analysis of B cell subsets by flow cytometry revealed that marginal zone B cells, which are thought to contain a potent regulatory subset, were targeted in the spleen (Figure 1, B and C) (32). Anti-CD45RB treatment leads to a B cell dependent expansion of CD4^+^Foxp3^+^ Tregs to promote long-term tolerance induction (22, 31). We hypothesized that this B cell mobilization would expand CD4^+^Foxp3^+^ Tregs in both SLE123 and B6 mice. An analysis of the Foxp3^+^ and CD25^+^ fractions of the CD4^+^ population revealed expansion of Tregs in both B6 and SLE123 mice following anti-CD45RB therapy (Figure 1, D and E).

### Anti-CD45RB uncovers inappropriate effector responses to tolerogenic signaling.

**Figure 2:**
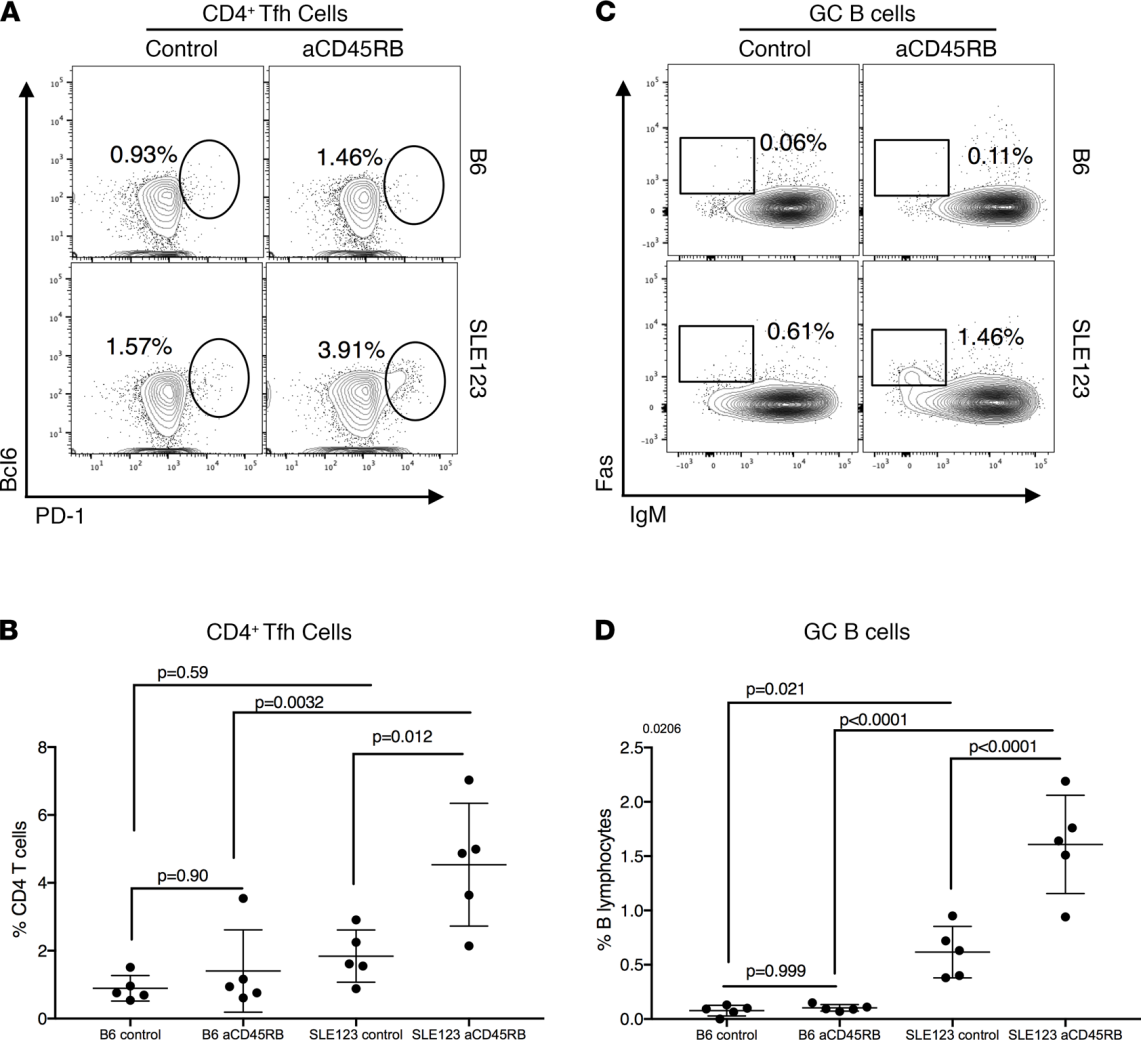
Effector CD4^+^ T cells respond inappropriately to anti-CD45RB, accelerating pathologic GC formation in young SLE mice. (**A** and **B**) A 7-day course of aCD45RB was administered to B6 and SLE123 mice. SLE123 mice experienced an increase in Tfh cells in response to aCD45RB. Quantified in **B**. (**C** and **D**) We hypothesized that this increase would also drive expansion of GC B cells, responsible for producing anti-nuclear antibodies in SLE. Flow cytometric analysis revealed aCD45RB-treated SLE123 mice expanded GC B cells, indicating an inappropriate response to aCD45RB therapy. Quantified in **D**. Analyzed using a 1-way ANOVA followed by Tukey’s multiple comparison test. All mice in **A**–**D** were female and 9–12 weeks of age, *n* = 5 per group.

While expansion of Tregs is important for long-term tolerance in anti-CD45RB treatment, the effector compartment must also be temporarily modulated to facilitate adequate regulation (33, 34). In healthy B6 mice, anti-CD45RB inhibits the germinal center (GC) response and temporarily cripples antibody production (27, 34). In clinical SLE and in the mouse model of disease, there is an expansion of T-follicular helper (Tfh) cells and GC B cells that collaborate to generate autoantibodies that occlude and damage the nephrons of the kidneys. We assessed the ability of anti-CD45RB therapy to modulate this interaction in SLE123 and B6 mice. Surprisingly, administration of anti-CD45RB to young SLE123 mice expanded both Tfh and GC B cells, suggesting that the expanded regulatory cells did not effectively control this process (Figure 2, A–D). In agreement with previous work, anti-CD45RB did not expand either Tfh or GC B cells in B6 mice (34). The expansion of regulatory B and T cell subsets in SLE123 mice along with the concomitant expansion of deleterious effector populations suggested competent regulatory cell targeting by treatment but an abnormal and regulation-resistant effector response. These data agree with previous observations by our lab that SLE123 mice possess functional regulatory cells but have effector cells that respond inappropriately to tolerizing signals (27).

### Anti-CD45RB alters metabolism in CD4^+^ T cells from B6 mice but not SLE123.

**Figure 3:**
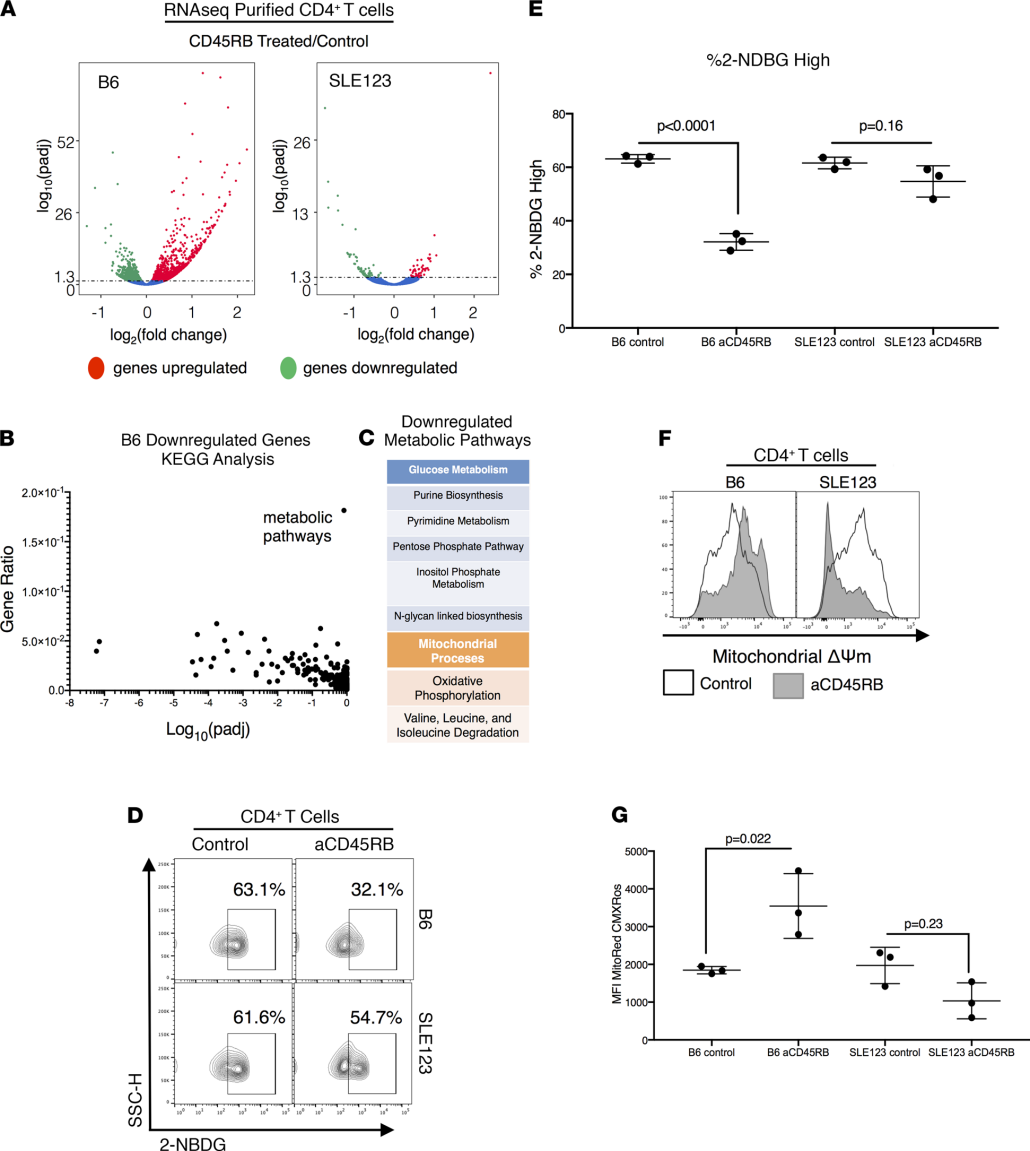
Anti-CD45RB drives transcriptional changes in metabolism in B6 CD4^+^ T cells, which are resisted by SLE123 mice. (**A**) CD4^+^ T cells were isolated from B6 and SLE123 mice that had been treated with a standard 7-day course of aCD45RB or left untreated (*n* = 3, 9- to 12-week-old female SLE or B6 mice). RNA-Seq was performed and analyzed for differential gene expression. The volcano plot demonstrates regulation of a subset of genes in response to aCD45RB only in B6 mice, while SLE123 mice largely resisted transcriptional changes. (**B**) KEGG analysis of the downregulated genes in B6 mice revealed metabolic pathways to have the highest gene ratio of regulated pathways. (**C**) Genes in this KEGG term could be classified broadly into 2 categories that impact both glucose metabolism and mitochondrial processes. (Full gene list available in Supplemental Figure 1B.) (**D** and **E**) Analysis of glucose uptake using a fluorescent glucose analog, 2NBDG, revealed a decrease in glucose uptake in CD4^+^ T cells only from B6 mice treated with CD45RB, which was resisted by SLE123 mice. This corroborated the observation of downregulation of genes associated with glucose metabolism. The percentage of CD4^+^ T cells with high glucose uptake is quantified in **E**. (**F** and **G**) Mitochondrial membrane potential, a measure of mitochondrial activity, was assessed in both B6 and SLE123 CD4^+^ T cells by flow cytometry utilizing MitoredCMXRos. This analysis revealed increased mitochondrial membrane potential (Δψ) in CD4^+^ T cells from B6 mice treated with aCD45RB. SLE123 mice resisted these changes and demonstrated a decrease in membrane potential. The median fluorescence intensity of MitoredCMXRos from **F** is quantified in **G**. The analyses in **D**–**G** were repeated at least 7 times with 3 or more 9- to 12-week-old female SLE123 or B6 mouse replicates in each group. Analyzed using a 1-way ANOVA and Tukey’s multiple comparison test.

We sought to understand how anti-CD45RB modulates the effector compartment, and therefore we compared the transcriptional profile via RNA-Seq of CD4^+^ T cells from B6 and SLE123 CD4^+^ T cells either treated with anti-CD45RB or left untreated. Comparative analysis demonstrated minimal change in SLE123 CD4^+^ T cells treated with anti-CD45RB as compared with untreated SLE123 controls; the response in B6 CD4^+^ T cells was significantly more robust and included multiple genes (Figure 3A; principal component analysis [PCA] in Supplemental Figure 1A; supplemental material available online with this article; https://doi.org/10.1172/jci.insight.143245DS1). Analysis of the responsive genes in B6 CD4^+^ T cells using KEGG terms revealed downregulation of multiple metabolic genes (Figure 3B; Full KEGG and GO analysis in Supplemental Figures 2 and 3). The genes contained in this KEGG pathway included glycolytic and mitochondrial pathways. The glucose-related pathways included N-glycan–linked biosynthesis, inositol phosphate metabolism, and downregulation of nucleotide biosynthesis (Figure 3C). Downregulation of mitochondrial processes, such as the breakdown of valine, leucine, and isoleucine important for mTOR signaling and fueling the Krebs cycle, was also observed. Importantly, there was downregulation of genes associated with mitochondrial OXPHOS. The full gene list can be found in Supplemental Figure 1B.

Reduction in transcription of genes in the pentose phosphate and N-glycan–linked biosynthesis pathways suggest alterations in glucose metabolism. We predicted that this change would lead to a decrease in glucose uptake among CD4^+^ T cells from B6 mice treated with anti-CD45RB. Analysis of isolated splenic CD4^+^ T cells revealed a reduction in glucose uptake among CD4^+^ T cells in B6 mice treated with anti-CD45RB, as measured by the uptake of fluorescent glucose analog 2-NBDG (Figure 3, D and E). In agreement with their resistance to transcriptional changes, SLE123 CD4^+^ T cells treated with anti-CD45RB resisted downregulation of glucose uptake (Figure 3, D and E). We did not observe differences in the rate of glycolysis or expression of glucose transporter Glut1 after anti-CD45RB, as measured by the extracellular acidification rate (Supplemental Figure 1, C and D). This observation was unsurprising as most cells were naive CD4^+^ T cells that utilize glycolysis sparingly (35).

RNA-Seq analysis revealed changes in nonlactate-producing glucose utilization pathways. These data indicate that a decrease in glucose uptake was most likely related to decreased demand via nucleotide precursor biosynthesis pathways and protein glycosylation. Untargeted metabolomics analysis of anti–CD45RB-treated CD4^+^ T cells from B6 mice affirmed the role of this nucleotide pathway, revealing a 16-fold increase in 6,8-dihydroxypurine (Supplemental Figure 1E). This covalently modified purine is often indicative of DNA damage and is characteristic of cells with depleted nucleotide precursors (36). Additionally, there was a marked reduction of nucleosides inosine and guanosine, which are produced downstream of the pentose phosphate pathway (Supplemental Figure 1E). The untargeted mass spectrometry data correlate well with the RNA-Seq data from anti–CD45RB-treated CD4^+^ T cells that indicate the downregulation of the pentose phosphate pathway, which is responsible for supporting nucleotide biosynthesis in naive CD4^+^ T cells.

Among the mitochondrial-targeted genes modulated, we identified changes in valine, leucine, and isoleucine metabolism, which are important metabolic contributors to the Krebs cycle. Additionally, we identified downregulation of genes associated with Complex I (MT-ND5, MT-ND6, MT-ND2, and MT-ND4) and Complex III (MT-CYTB) of the mitochondria (Supplemental Figure 1B). MitoTrackerCMXRos, which accumulates in the inner membrane of the mitochondria in response to increased membrane potential (Δψ), was used to measure changes in mitochondria function. B6 anti–CD45RB-treated CD4^+^ T cells possessed heightened mitochondrial membrane potential (hyperpolarized) compared with controls. SLE123 T cells presented with decreased Δψ in response to anti-CD45RB, indicating a divergent response as compared with B6 (Figure 3, F and G).

In times of nutrient deficiency and mitochondrial dysfunction, mitochondrial maintenance through mitophagy is reduced, leading to large fused mitochondria (37). To assess the emergence of enlarged mitochondria with anti-CD45RB treatment, mitochondrial mass was analyzed via MitoTracker Green, which labels mitochondria regardless of the membrane potential, via flow cytometry. Mitochondrial size was increased in B6 CD4^+^ T cells treated with anti-CD45RB, but no such change occurred in SLE123-treated mice (Supplemental Figure 1, F and G). Overall, these data demonstrate that anti-CD45RB targets both glucose and mitochondrial metabolism in B6 mice; this metabolic shift is resisted by SLE123 CD4^+^ T cells.

### Anti-CD45RB targets metabolic genes through a CREB/ATF-1 pathway component that is sensitive to cyclosporine A.

**Figure 4:**
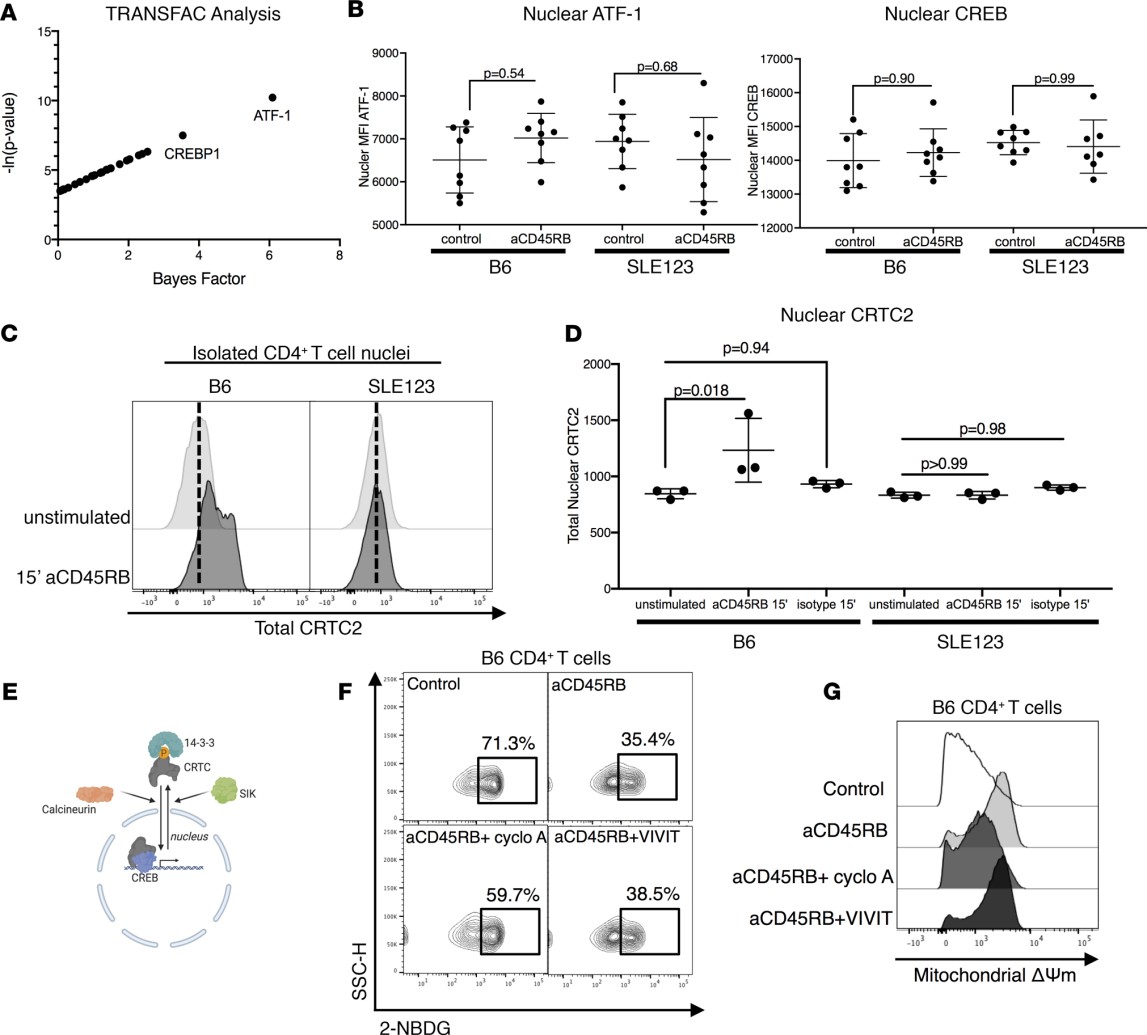
A cyclosporine sensitive element modulates immune changes seen with aCD45RB and is defective in SLE123 CD4^+^ T cells. (**A**) TRANSFAC analysis of the metabolic genes regulated by aCD45RB in B6 mice revealed ATF-1 and CREBP likely regulated these genes. (**B**) As ATF-1 and CREB share similar binding motifs, we assessed the nuclear import of both CREB and ATF-1 in aCD45RB-treated and untreated CD4^+^ T cells from B6 and SLE. Briefly, nuclei from purified CD4^+^ T cells were extracted and stained for total CREB and ATF-1. The nuclear localized amount of each protein was measured by flow cytometry. No significant difference was noted in any of the conditions regardless of strain or treatment. *n* = 8 per group, 9- to 12-week-old female mice. (**C** and **D**) The transcriptional activity of CREB can be modified by translocation of CRTCs. Nuclear staining demonstrated translocation of CRTC2 to the nucleus following 15 minutes of aCD45RB stimulation only in isolated nuclei from B6 CD4^+^ T cells. SLE123 mice did not demonstrate the same response. Quantified in **D**. Representative of 3 repeats with at least 3 biologic replicates per group, utilizing 9- to 12-week-old female mice. (**E**) The translocation of CRTC proteins is controlled by the phosphatase calcineurin and the SIK, which control nuclear import and export respectively. (**F** and **G**) Pretreatment of B6 CD4^+^ T cells with calcineurin inhibitor, cyclosporine A, but not the NFAT-specific inhibitor, VIVIT (a major target of calcineurin), prevented the metabolic changes induced by aCD45RB. Cyclosporine prevented the downregulation of glucose uptake and changes in mitochondrial dynamics. The NFAT inhibitor VIVIT had no effect on the metabolic changes in glucose uptake or mitochondrial Δψ induced by aCD45RB. Analyzed by 2-way ANOVA and Tukey’s multiple comparison test. *n* = 5 per each treatment group, 9- to 12-week-old female mice.

We utilized the TRANScription FACtor (TRANSFAC) database to determine the transcription factors responsible for regulation of these metabolic genes in response to anti-CD45RB to further define the mechanism and to potentially find a druggable transcription factor to target in SLE123 mice (38). This analysis revealed that changes in the CREB pathway including CREBP1 and ATF-1 (a transcription factor with similar structure and function to CREB) may be responsible for this change (Figure 4A). To determine whether anti-CD45RB modulated CREB or ATF-1, we isolated CD4^+^ T cells from B6 and SLE123 mice treated with anti-CD45RB or left untreated. We then isolated the nuclei of purified CD4^+^ T cells and stained for total CREB and total ATF-1. We did not detect an increase in the nuclear localization of ATF-1 or CREB (Figure 4B). The activity of CREB can be modulated by a class of CREB-regulated transcriptional coactivators (CRTCs) that are sequestered outside of the nucleus by the activity of salt-inducible kinase (SIK) (39). Calcineurin, the target of cyclosporine A, dephosphorylates these CRTCs and allows them to translocate to the nucleus and interact with CREB (Figure 4E). CD4^+^ T cells from B6 mice were incubated with anti-CD45RB (20 μg/mL) over a course of time with or without cyclosporine A. Only CD4^+^ T cells that were stimulated with anti-CD45RB in the absence of cyclosporine A demonstrated decreases in CRTC phosphorylation (Supplemental Figure 4, A and B). Based on these data, we hypothesized that treatment with anti-CD45RB would induce CRTC translocation in CD4^+^ T cells. Isolation of nuclei from CD4^+^ T cells of B6 or SLE123 stimulated with anti-CD45RB for 15 minutes indicated that CRTC2 only translocated to the nucleus in B6 CD4^+^ T cells, not in SLE123 T cells (Figure 4, C and D, and Supplemental Figure 4C). The signaling studies affirmed the relationship of the metabolic abnormalities to a cyclosporine A–sensitive pathway, which is also a requisite for tolerance induction by anti-CD45RB (40). Based on these data, we hypothesized that blocking the activity of calcineurin and, by extension, CRTC, translocation to the nucleus would inhibit metabolic changes induced by anti-CD45RB. B6 mice were treated with cyclosporine A and anti-CD45RB, and glucose uptake and mitochondrial Δψ were measured via flow cytometry (Figure 4, F and G). Cyclosporine A prevented changes in metabolism induced by anti-CD45RB, suggesting a mechanistic association with tolerance induction. In addition to its role in regulation of CRTCs and the CREB pathway, calcineurin also dephosphorylates the nuclear factor of activated T cells (NFAT) to allow nuclear activity of this protein. NFAT has also been implicated in the control of immune regulation and metabolism (7, 26, 41–44). We assessed whether NFAT contributed to the metabolic changes induced by anti-CD45RB. We utilized the NFAT-specific competitive peptide, VIVIT, to inhibit the ability of calcineurin to activate NFAT (45, 46). The specificity of this peptide inhibitor left the broader phosphatase activity of calcineurin intact. Treatment of CD4^+^ T cells from B6 mice with VIVIT and anti-CD45RB together did not inhibit the metabolic changes characteristic of anti-CD45RB therapy, making it unlikely that NFAT is the major target for anti–CD45RB-induced metabolic changes in T cells (Figure 4, F and G).

Simultaneous delivery of anti-CD45RB and calcineurin inhibitor cyclosporine A inhibits induction of transplantation tolerance in murine models (40). This pathway appears to regulate metabolic shifts through modulation of CREB/CRTCs, which are known to be dysregulated in SLE123, rather than NFAT. Unfortunately, there is no evident approach to restore normal CRTC activity therapeutically.

### Extracellular glycosylation and CD45RB expression patterns on CD4^+^ T cells are altered in SLE123 mice.

**Figure 5:**
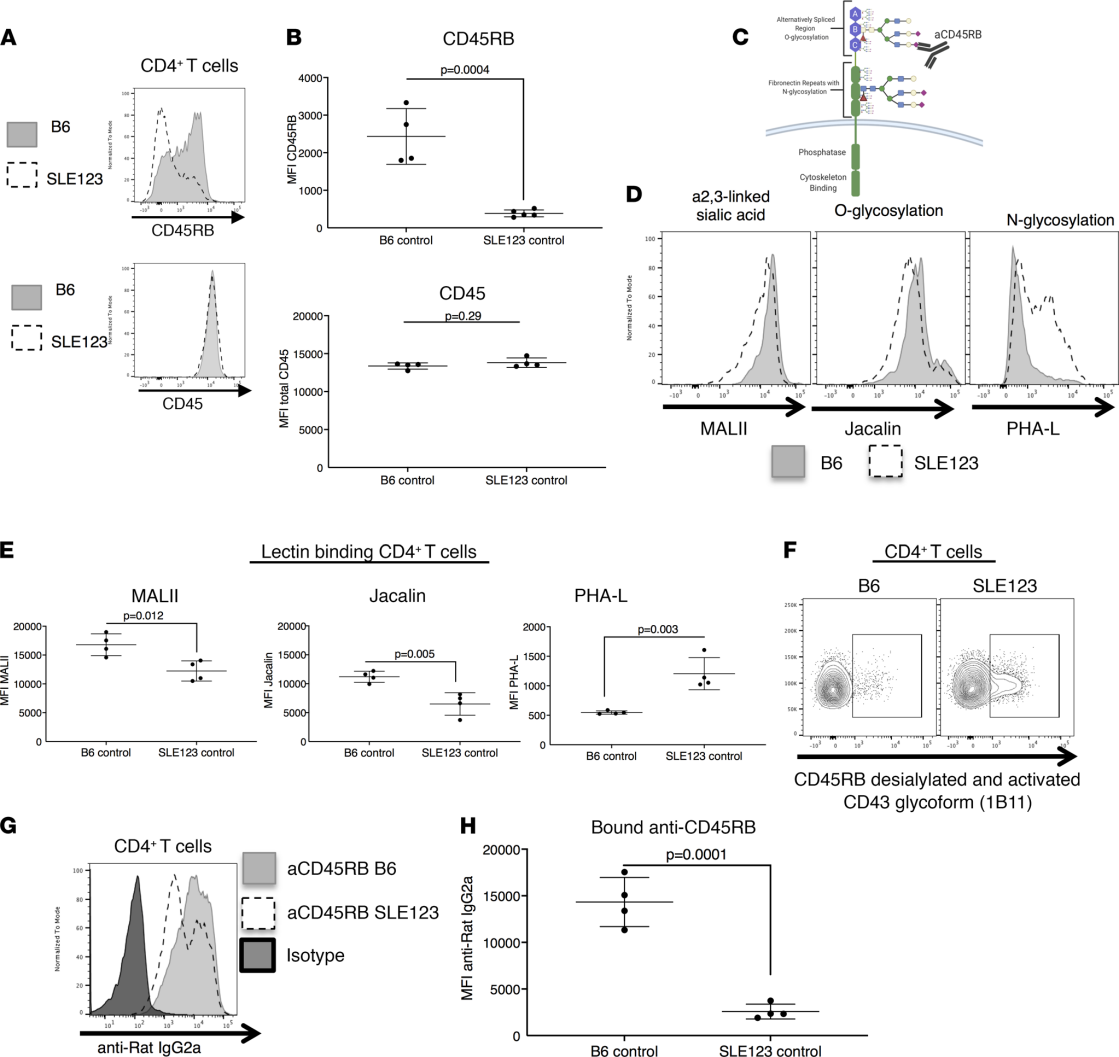
CD4 surface expression of CD45RB and global glycosylation are altered in SLE123 mice. (**A** and **B**) Cell surface expression of CD45RB and total CD45 were measured by flow cytometry on CD4^+^ T cells. SLE123 demonstrated a downregulation of CD45RB on the cell surface as compared with B6 CD4^+^ T cells. There was no difference in pan-CD45 expression. Quantified in **B**. (**C**) CD45 is composed of an intracellular region that controls cytoskeletal binding and its phosphatase activity. The extracellular domain is composed of a region with fibronectin repeats that is heavily N-glycosylated. An alternatively spliced region is heavily O-glycosylated, and this region imparts unique functions to each CD45 isoform. Additionally, a sialic acid residue on the B portion of this region is essential for the binding of therapeutic aCD45RB. (**D** and **E**) We utilized lectins to detect the level of α-2,3–linked sialylation (MALII), O-linked glycosylation (Jacalin), and N-linked glycosylation (PHA-L) in CD4^+^ T cells from B6 and SLE123 mice. We determined SLE123 CD4^+^ T cells had reduced levels of α-2,3–linked sialic acids and O-glycosylation and an increase in N-glycosylation. Quantified in **E**. (**F**) Utilizing an antibody that detects a desialylated form of anti-CD45RB, we determined SLE123 CD4^+^ T cells possessed increased binding of this antibody compared with B6. (**G** and **H**) To determine the binding of therapeutic aCD45RB to B6 and SLE123 CD4^+^ T cells, we incubated splenocytes from both strains with aCD45RB, followed by an anti–rat IgG2a antibody conjugated to phycoerythrin. Flow cytometry demonstrated CD4^+^ T cells from SLE123 mice had reduced binding of the therapeutic antibody. Quantified in **H**. Analyzed using a Student’s *t* test. Representative data of at least 4 experimental repeats, with at least 3 biologic replicates of 9- to 12-week-old mice in each group.

The CREB and CRTC signaling pathways are strongly controlled by glucose and the activity of metformin target AMPKa, and in turn AMPKa regulates both mitochondrial and glucose metabolism (39). Additionally, the trafficking, surface expression, and protein-protein interactions of the CD45 protein are heavily regulated by glycosylation and its expression is required for anti-CD45RB activity (47, 48). We hypothesized that surface expression of CD45RB in SLE123 mice may be deficient in response to changing T cell metabolism, thus preventing binding of the therapeutic antibody. We assessed cell surface expression of CD45RB on CD4^+^ T cells from B6 and SLE123 mice. CD45RB on the surface of SLE123 CD4^+^ T cells was decreased compared with B6 CD4^+^ T cells (Figure 5, A and B). Interestingly, we did not detect a decrease in pan-CD45 expression on CD4^+^ T cells in SLE123 mice (Figure 5, A and B). CD45 contains several domains, of which the intracellular domain controls cellular signaling and cytoskeletal interactions. The extracellular domain is composed of a region of fibronectin repeats that is heavily N-glycosylated and an alternatively spliced region that is heavily O-glycosylated (47). Alternative splicing imparts unique functionality on each individual CD45 isoform (20, 49). The therapeutic antibody targets the B segment of the alternatively spliced region. The binding of therapeutic aCD45RB is dependent on proper glycosylation and end sialylation (Figure 5C). We tested whether lectins that bind and block specific glycosylation motifs would block the binding of aCD45RB. Indeed, only those lectins that blocked α-2,3–linked sialylation and O-glycosylation inhibited the binding of anti-CD45RB (Supplemental Figure 5). We determined whether altered metabolism in CD4^+^ T cells from SLE123 altered the cell surface levels of O-glycosylation and sialylation. Utilizing fluorescently tagged Maackia amurensis lectin II (MALII) to detect α-2,3–linked sialylation and Jacalin to detect O-glycosylation, we determined that SLE123 CD4^+^ T cells possessed reduced surface levels of both (Figure 5, D and E). In turn, we noted an increase in global CD4^+^ T cell N-glycosylation in SLE123, using FITC-labeled lectin Phaseolus vulgaris leucoagglutinin (PHA-L) (Figure 5, D and E). We also noted binding of an antibody that detects desialylated CD45RB, and an activated glycoform of CD43 (1B11) was increased, further affirming a shift in post-translational modification of CD45RB (Figure 5F) (50). We predicted these changes would decrease binding of anti-CD45RB to the surface of SLE123 CD4^+^ T cells. We incubated splenocytes with anti-CD45RB or an isotype control antibody and utilized a fluorescently tagged anti–Rat IgG2a to detect its presence on the surface of CD4^+^ T cells. We noted decreased binding of anti-CD45RB on the surface of CD4^+^ T cells in SLE123 CD4^+^ T cells compared with B6 CD4^+^ T cells (Figure 5, G and H). Taken together, these data suggest that modifications in intracellular metabolism alters the glycosylation patterns and expression of therapeutic target CD45RB, which may then prevent tolerance induction and may also reflect a general change in protein trafficking and expression.

### 2DG and metformin combination treatment improves anti-CD45RB binding, restores metabolic modulation, and eliminates pathogenic cell subsets.

**Figure 6:**
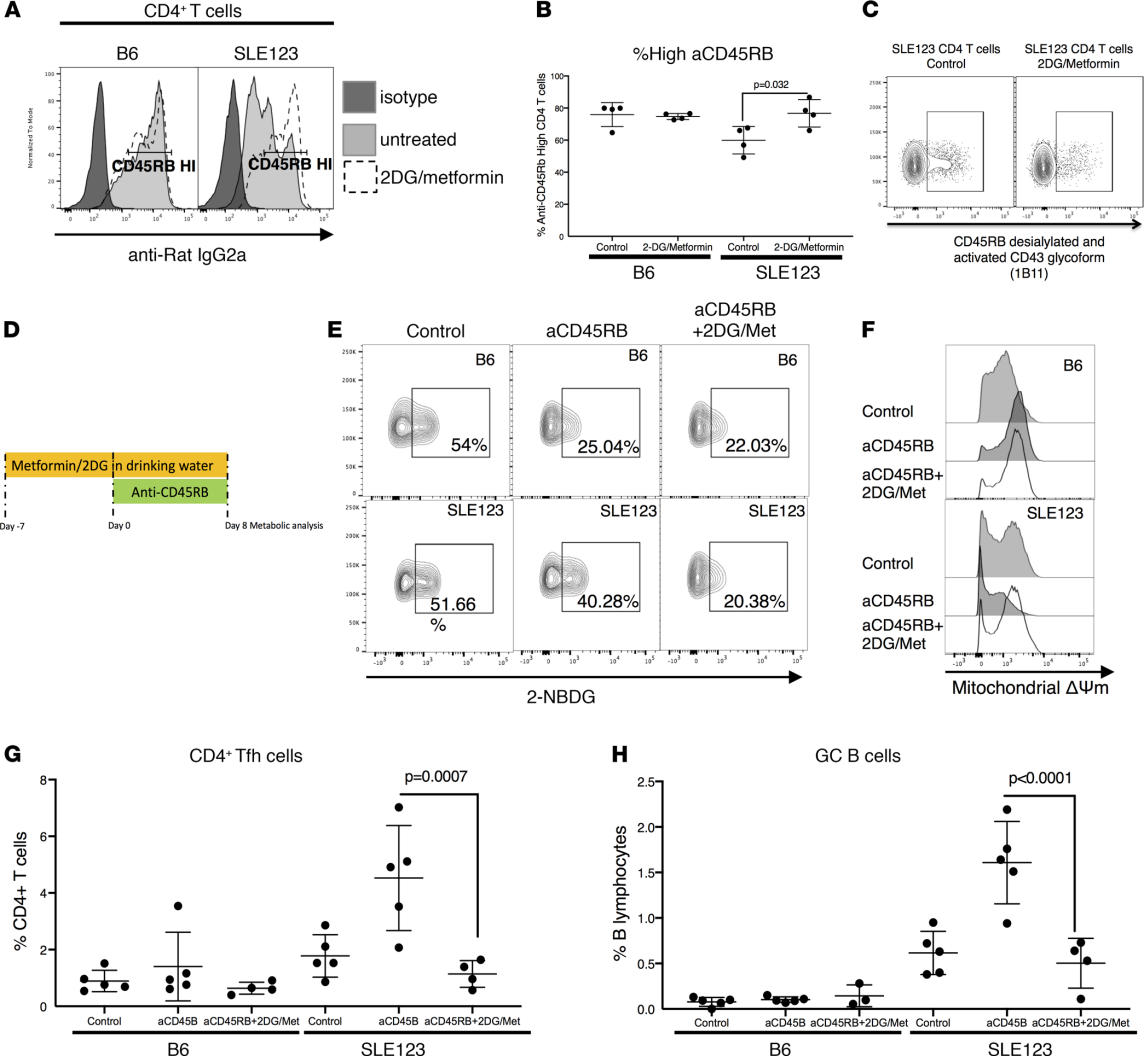
Metabolic intervention improves aCD45RB binding and restores responsiveness to aCD45RB therapy. (**A** and **B**) We predicted treatment of SLE123 mice with 2DG and metformin for 1 week would improve the binding of aCD45RB to CD4^+^ T cells. Treatment with these metabolic therapies improved the binding of aCD45RB (dotted line) as compared with untreated controls (gray histogram). Quantified in **B**. Representative of 2 experimental repeats, with at least 4 biologic replicates of 9- to 12-week-old female mice in each group. (**C**) Treatment of SLE123 mice with 2DG/Met reduced the binding of antibody 1B11 that detects desialylated CD45RB. (**D** and **F**) B6 and SLE123 mice were treated with 2DG/Met for 1 week before starting aCD45RB and continued for an additional week concomitant with aCD45RB. At the end of this treatment, mice were sacrificed, and metabolic parameters and cell subsets were analyzed. (**E**) Treatment with metabolic modulators + aCD45RB restored the metabolic regulation by aCD45RB in SLE123 CD4^+^ T cells. (**G** and **H**) This triple therapy also reduced the expansion of Tfh and GC B cells. Analyzed using a 1-way ANOVA followed by Tukey’s multiple comparison test. In **D**–**H**, *n* = 5, 9- to 12-week-old female mice per group. (Control and aCD45RB-treated mice are also shown in Figure 2, B and D.)

We hypothesized that metabolic modulation would normalize glycosylation to restore the binding of anti-CD45RB. Because glycosylation is driven by both glucose metabolism and OXPHOS and both processes are abnormal in SLE123 mice, we treated SLE123 and B6 mice with the competitive glycolysis inhibitor 2DG and OXPHOS inhibitor metformin via their drinking water for 2 weeks. Analysis of treated mice revealed improvement in anti-CD45RB binding on SLE123 CD4^+^ T cells (Figure 6, A and B). Additionally, there was a reduction in binding of the antibody (1B11) that detects desialylated CD45 as well as the activated glycoform of CD43 (Figure 6C).

After determining that metabolic modulation restores the ability of anti-CD45RB to bind CD4^+^ T cells in SLE123, we hypothesized that this change would be sufficient to restore responsiveness to anti-CD45RB as observed in B6 mice. Mice were treated with 2DG and metformin in their drinking water 7 days before beginning a standard course of anti-CD45RB; the metabolic treatment was continued until the end of the anti-CD45RB treatment course (Figure 6D). We determined that metabolic therapy in combination with anti-CD45RB restored regulation of metabolism. CD4^+^ T cells from SLE123-treated mice displayed reduced glucose uptake, increased mitochondrial mass, and mitochondrial membrane potential, similar to B6 mice (Figure 6, E and F). We predicted this restoration of metabolic modulation would also correct the expansion of pathologic cell subsets seen in SLE123 with anti-CD45RB alone. Assessment of Tfh cells and GC B cells by flow cytometry revealed reduction in both subsets after treatment with metabolic modulation plus anti-CD45RB (Figure 6, G and H). As previously demonstrated (Figure 2, A–D), anti-CD45RB alone exacerbates these pathologic cell subsets. These data suggest that proper cellular metabolism and responsiveness are prerequisites for the therapeutic application of anti-CD45RB.

### Metabolic therapy combined with anti-CD45RB reduces lupus pathology and promotes allograft acceptance.

**Figure 7:**
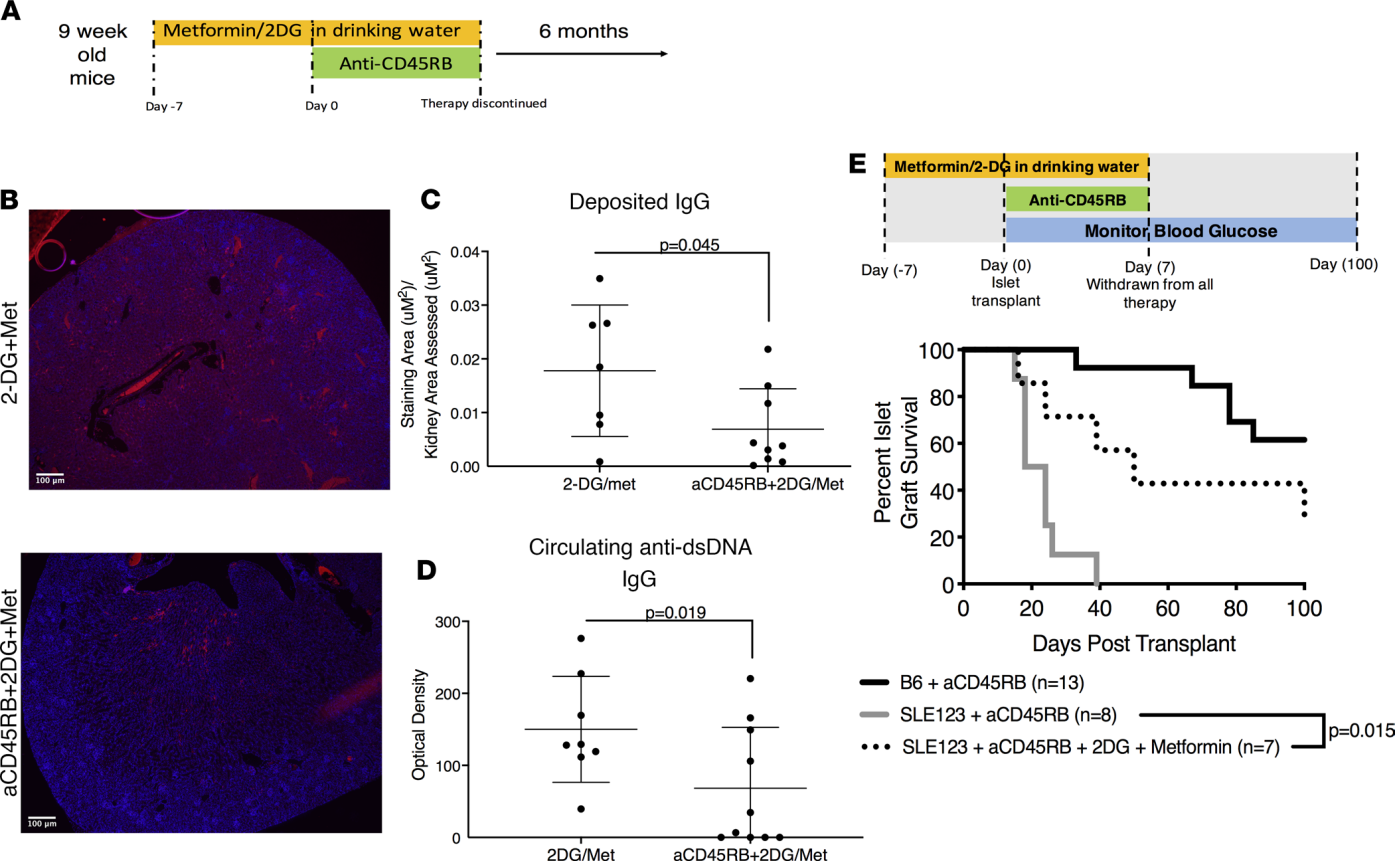
Metabolic intervention improves lupus pathology and tolerance to transplanted islets. (**A**) To determine whether a short therapeutic course of aCD45RB combined with 2DG/Met would improve the pathology of SLE123 mice, we treated 9-week-old mice with 2DG/met for 1 week before a standard course of aCD45RB with continued 2DG/Met. We also had a group that received 2DG/Met alone for 2 weeks. At that point, treatment was discontinued, and mice were allowed to age for 6 months before they were sacrificed and analyzed. (**B** and **C**) Kidney sections from SLE123 mice treated with triple therapy or 2DG/Met alone were stained with anti-IgG (red). There was a notable reduction in IgG deposition in mice treated with 2DG/Met and aCD45RB as compared with metabolic intervention alone. The area of red staining was quantified in these sections as shown in **C** and analyzed using a Student’s *t* test. (**D**) Additionally, we assessed the amount of circulating anti-dsDNA IgG. We noted a decreased in those mice treated with the triple therapy regime as compared with 2DG/Met alone. Analyzed using χ^2^ test. (**E**) To assess the capacity for durable tolerance with this triple therapy, we utilized an islet transplant model. Male and female mice 9–12 weeks old were placed on 2DG/Met 1 week before grafting MHC-disparate C3H islets under the kidney capsule. These mice received aCD45RB before metabolic therapy was discontinued. The blood glucose was tracked in these mice, as compared with B6 mice with aCD45RB or SLE123 mice that only received aCD45RB. Rejection was scored by 2 consecutive blood glucose readings of greater than 200 mg/dL. There is an increase in islet survival in SLE123 mice that received the triple therapy as compared with mice that only received aCD45RB. (B6 + aCD45RB, *n* = 13; SLE123 + aCD45RB, *n* = 8; and SLE123 + aCD45RB + 2DG + Metformin, *n* = 7). Scale bars: 100 μm.

We hypothesized that this triple therapy would correct faulty tolerance mechanisms in SLE123 mice. To assess this hypothesis, we utilized the same 2-week triple therapy in 9-week-old SLE123 mice (Figure 7A). Six months after the initial 2-week intervention, approximately 50% of SLE123 mice demonstrated an overall reduction in IgG deposition in the kidneys as scored by fluorescence staining (Figure 7, B and C). Additionally, there was a reduction in overall circulating anti-dsDNA IgGs in mice treated with the single course of triple therapy (Figure 7D). Mice transplanted with C3H islets and treated with the triple therapy demonstrated improved islet survival and long-term graft acceptance compared with anti-CD45RB alone (Figure 7E). Overall, these results demonstrate that metabolic inhibition coupled with signaling driven by anti-CD45RB improves tolerance and disease outcome in SLE123 mice.

## Discussion

This study reveals a critical role for metabolism as a modifiable barrier to T cell tolerance induction in SLE123 mice. The importance of glucose and mitochondrial metabolism in T cell function has been borne out repeatedly in murine and human studies, but whether targeting metabolism is simply immune suppressive or fundamentally alters the capacity for immune tolerance induction has not been established. During T cell activation, OXPHOS increases and is required for efficient and complete activation (7). Similarly, during activation and effector function, glucose is shunted to the glycolytic pathway, a phenomenon termed the Warburg effect (6). This metabolic switch allows for byproducts and intermediates of glycolysis to be utilized for cell division and production of molecules for proper effector function. CD4^+^ T regulatory cells are thought to utilize lipids and mitochondrial OXPHOS to mediate their function, although studies are emerging that challenge that paradigm (51–53). This putative difference allows the use of metabolic inhibitors to target effector metabolism while leaving Treg metabolism and function mostly intact.

Our results demonstrate that metabolism is a fundamental target of tolerance-inducing therapy and, hence, changes in the metabolic condition of T cell targets form a barrier to successful immune therapy. Specifically, abnormal metabolism in SLE123 mice leads to alteration of the glycoprotein structure of CD4^+^ T cells. In CD4^+^ T cells from SLE123 mice, N-glycosylation was increased and both O-glycosylation and α-2,3–linked sialylation were decreased. CD45 is a heavily edited molecule both via splicing and differential O- and N-glycosylation. These modifications are of particular importance to anti-CD45RB therapy, as this clone (MB23G2) reacts specifically with a sialylated moiety located on an O-glycosylated portion of CD45RB (54). Enhanced N-glycan branching potentially inhibited the ability of our therapeutic antibody to bind to its target. Previous literature indicates that SLE123 CD4^+^ T cells demonstrate enhanced glucose metabolism (9). This enhanced metabolism could lead to changes in the glycosylation of CD45, CD25 (IL2RA), or cell-adhesion molecules like CD2 and CD48, due to the availability of N-glycan precursors. Because many proteins depend on glycosylation for their normal function, changes in glycosylation will impact intracellular processes as well as cell-cell interactions that are important for normal T cell function. T cells derived from samples from patients with SLE also confirm these altered glycosylation patterns (55).

Studies in healthy B6 mice indicated that tolerance-inducing therapy anti-CD45RB targeted and downregulated both glucose and mitochondrial metabolism. SLE123 did not experience this same modulation, presumably because of the inability of anti-CD45RB to bind and induce an appropriate signal. How treatment with anti-CD45RB modulates signaling to induce tolerance is not known despite its extensive track record in transplantation in mice and nonhuman primates (23, 56, 57). The role of mTOR and AKT signaling has not been fully understood in transplant tolerance induced by anti-CD45RB. Even though we observed changes in components important for mTOR signaling, we did not observe changes in mTOR signaling in T cells after anti-CD45RB (data not shown). Neither did we observe enhanced mTOR signaling as has been previously reported. Our analysis was conducted at a much earlier age than what has been reported; therefore, it remains possible that such differences could be provoked under the right conditions or that the differences at a young age are too small to detect with the methods we employed.

The opportunity to compare tolerance-sensitive and resistant animals on the same overall genetic background presented a new avenue to resolve anti-CD45RB signaling. The ability of anti-CD45RB to induce tolerance in CD4^+^ T cells has been known to rely on a cyclosporine-sensitive pathway (40). While this prior evidence would suggest that NFAT is the target of anti-CD45RB, use of a specific peptide inhibitor of NFAT did not prevent metabolic changes induced by anti-CD45RB. Evaluation of the RNA-Seq data revealed the metabolic pathways modified by anti-CD45RB are targets of the CREB/ATF-1 transcription pathway. Anti-CD45RB may induce the nuclear localization of CREB/ATF-1 cofactors of transcription (CRTC2) by dephosphorylating it, as suggested by our data. This dephosphorylation is regulated by calcineurin, which is also a target of cyclosporine A. CREB abnormalities have been extensively documented in SLE123 mice, including reduced activity and abnormal function of the CREB- and cAMP-responsive element binding modulator (CREM), an alternative splicing isoform of CREB with capacity to inhibit CREB transcriptional activity (58). Additionally, abnormal nuclear activity of CamKIV in T cells from patients with lupus leads to binding of CREM to the IL-2 promoter, inhibiting transcription of this gene (59, 60). This interaction leads to abnormal T cell function and possible Treg abnormalities; further studies are needed to determine how the large CREB network controls immune tolerance decisions.

This work demonstrates that metabolism plays an important role in regulating cell surface proteins important for proper T cell function. This observation has implications not only for intrinsic cellular function but also for cell-cell communication, which is essential for immune regulation. In a broader sense this study demonstrates that targeting metabolism provides a window for tolerance to be established. While studies are focusing largely on utilizing therapeutics that target metabolism singly, effectively providing immune suppression by limiting access to nutrients, it may be important to investigate combining metabolic therapies with other immune therapies to improve their efficacy. Metabolism plays important roles in regulation of other extracellular molecules currently targeted by immune therapy in autoimmune disease in autoimmunity and transplant, including CD40, CD20, CTLA-4, CD25, and CD3. Additionally, metabolites can alter intracellular signaling processes via production of reactive-oxygen species or regulation of biomolecules that participate in signaling pathways. This interaction highlights the need to understand the metabolic state of targets cells to determine the best immune therapy or whether “metabolic preconditioning” is required for maximum effectiveness.

Phenotypic analysis of anti–CD45RB-induced cell phenotypes in SLE123 and B6 mice illustrate several important phenomena characteristic of effective versus ineffective tolerogenic responses. First, it has been clearly demonstrated that metabolic inhibition facilitates a reduction in the GC response in lupus (9, 11, 12, 61–63). Our data indicate that normally tolerogenic signaling exacerbates pathologic cell subset expansion on the SLE123 background even in young mice prior to the spontaneous emergence of this disease-associated phenotype. Thus, therapy that drives tolerance during normal immunity may enhance autoimmunity unless the system is preconditioned with a metabolic modulatory therapeutic. Second, mitochondrial hyperpolarization in immune cells is a feature of both murine and human lupus (16, 64). This hyperpolarization seems to be caused by chronic activation and production of metabolic byproducts such as reactive-oxygen species. When considering how to intervene with metabolic therapy in the future, we must carefully study whether long-term metabolic therapy may exacerbate the already metabolically taxed state of immune cells in lupus or whether it may prevent hyperactivation and quell metabolic strain by reduction of nutrient utilization in immune cells. Our study presents an alternative strategy in which a tolerizing therapy is combined with a brief metabolic intervention to provide lasting protection. Future studies need to consider whether these short windows of metabolic intervention coupled with immune therapy should be repeated to provide maximal protection from symptomatic relapse.

## Methods

### Animals.

C3H (C3H/HeJ), SLE123(B6;NZM-*Sle1^NZM2410/Aeg^ Sle2^NZM2410/Aeg^ Sle3^NZM2410/Aeg^*/LmoJ), and B6 (C57BL/6J) were purchased from The Jackson Laboratory. SLE123 mice were developed by Edward Wakeland (13). Mice were housed in a specific-pathogen–free facility at Vanderbilt University.

### Isolation of CD4^+^ T cells and RNA-Seq analysis.

CD4^+^ T cells were isolated by negative magnetic cell sorting (MACS) from 9-week-old SLE123 or B6 mice, rendering a purity of approximately 98% CD4^+^ T cells. CD4^+^ T cells were further sorted into CD25^+^ and CD25^–^ via positive selection. These cells were frozen and sent to Novogene where they were sequenced, and bioinformatic analysis was carried out on the resulting data. Briefly, the raw data image file resulting from the sequencing was transformed to Sequenced Reads by CASAVA base recognition and stored as FASTQ format files. The data were quality control checked for GC content and error rate, and data were filtered to remove low-quality reads or those with adaptors. After mapping to a reference genome, gene expression analysis was determined by utilizing the fragments per kilobase of a transcript per million base pairs. Correlation analysis of biological replicates was carried out with a Pearson correlation coefficient cutoff above 0.92, followed by PCA. Differential expression analysis was carried out for 2 conditions at a time using the R package DESeq2 (65). Enrichment analysis was done using the clusterProfiler software (66) for both GO and KEGG enrichment with a cutoff of *P*_adj_ < 0.05. The data discussed in this publication have been deposited in NCBI’s Gene Expression Omnibus (GEO) and are accessible through GEO Series accession GSE181816 (https://www.ncbi.nlm.nih.gov/geo/query/acc.cgi?acc=GSE181816) (67).

### Tolerizing and metabolic treatment.

Anti-CD45RB (Bio X Cell, MB23G2) was given by intraperitoneal injection in a 100 μg dose on days 0, 1, 3, 5, and 7. Mice were given Metformin (Enzo Life Sciences) at 3 mg/mL and 5 mg/mL of 2DG (Tocris) dissolved in their drinking water for 2 weeks. In instances where anti-CD45RB and metabolic treatment were combined, mice were given Metformin/2DG in the described dose for 1 week before beginning the standard anti-CD45RB regimen.

### Metabolic characterization of CD4 ^+^T cells.

Spleens were rendered to single-cell suspensions by crushing through a 70 μm filter. After isolation, cells were rested in DMEM supplemented with 10% FBS (MilliporeSigma), Pen-Strep (Gibco), and 2-ME (Invitrogen) for 30 minutes. For analysis of glucose uptake with 2-NBDG (Thermo Fisher Scientific), cells were rested for 30 minutes in glucose-free DMEM prepared as indicated above and then fluorescent glucose analog was added. Then 2-NBDG (Life Technologies) was added at a final concentration of 30 μM from a stock solution. For analysis of mitochondria membrane size, MitoTracker Green (Thermo Fisher Scientific) was added in regular media at a final concentration of 30 nM. For measurement of mitochondrial Δψ, MitoRed CMXRos (Thermo Fisher Scientific) was used at a concentration of 100 nM. Both were incubated for 1 hour and then washed with PBS containing 3% FBS and Pen-Strep. All samples were then stained for extracellular markers and analyzed by flow cytometry. When analyzing mitochondrial dynamics, sodium azide was eliminated from staining buffers due to its toxic effects on mitochondria function.

### Metabolomics analysis.

CD4^+^ T splenocytes from the spleens of B6 mice treated with anti-CD45RB or left untreated and SLE123 mice treated with anti-CD45RB or left untreated were isolated and separated into subsets via MACS. Supernatants were removed and cell pellets were flash-frozen using a dry ice/ethanol bath. Samples were then placed at –80°C for storage. A “blank” sample was included, which consists of the residue of 3 mL of column wash (MACS buffer: prepared in-house Pen-Strep, 3% FBS diluted in 1x PBS from Corning) eluted into a tube and then washed twice with PBS. Cells were shipped to Mayo Clinic. Each sample was analyzed in duplicate via a 1200 Agilent UPLC that was connected to an Agilent 6500 Q-TOF-MS. Each sample was analyzed in both positive and negative modes of electron spray ionization for maximum coverage of metabolites in samples using both hydrophilic interaction chromatography (HILIC) and nonpolar reverse phase (C18) ultra-high pressure liquid chromatography (UPLC). Putative identification of metabolites was carried out against Metlin (68). To determine significance, an unpaired *t* test for 2 groups with multiple testing correction (Q < 0.05) was carried out.

### Nuclear isolation.

CD4^+^ T cells were isolated from B6 or SLE123 spleens via negative MACS. These cells were then rested in DMEM with 5% FBS, Pen-Strep, and 2-ME for 1 hour. They were then stimulated as described. These cells were then resuspended in a buffer containing 320 mM sucrose (Thermo Fisher Scientific), 10 mM HEPES buffer (Corning), 8 mM MgCl_2_ (Invitrogen), Roche EDTA-free Complete Protease Inhibitor, and 0.1% Triton X-100 (MilliporeSigma) for 15 minutes on ice. The cells were then fixed for 30 minutes on ice with 4% PFA (Pierce) added to the previously described buffer. Cells were then washed and permeabilized with 0.3% Triton X-100 on ice for 15 minutes. Isolated nuclei were then washed with FACS buffer containing 3% FBS and sodium azide (MilliporeSigma). They were then stained with SyTOX Green Nucleic Acid Stain (Thermo Fisher Scientific), and total CREB (Cell Signaling Technology, 48H2), total ATF1 (MilliporeSigma, SAB4501950), or total CRTC2 (MilliporeSigma, 5B10). Individual samples were acquired on a BD LSR Fortessa Flow Cytometer and analyzed with FlowJo software (TreeStar).

### Measuring phosphorylation of intracellular proteins.

Total splenocytes were isolated and rested in DMEM with 5% FBS, Pen-Strep, and 2-ME for 1 hour. They were then stimulated with 20 μg/mL of anti-CD45RB. Cells were then centrifuged and simultaneously fixed and permeabilized with Foxp3/Transcription Factor Staining Buffer Set (eBioscience) for 30 minutes. These cells were then stained with anti–phospho-CRTC (MilliporeSigma).

### Cellular phenotyping with flow cytometry.

Splenocytes were stained with fluorophore-conjugated antibodies: CD19 (6D5 BioLegend catalog 115530), B220 (RA3-6B2 eBioscience catalog 48-0452-82), Bcl-6 (K112-91 BD Biosciences catalog 561525), CD4 (RM4-5 eBioscience catalog 56-0042-82), CD8a (53-6.7 BD Biosciences catalog 557959), CD25 (7D4 eBioscience catalog 13-0252-82), CD95 (Jo2 BD Biosciences catalog 554257), CD44 (IM7 eBioscience catalog 48-0441-80), CD62L (MEL-14 BD Biosciences catalog 553152), Foxp3 (FJK-16s eBioscience catalog 53-5773-82), IgM (II/41 eBioscience catalog 17-5790-82), and PD-1 (J43 eBioscience catalog 13-9985-81). Individual samples were acquired on a BD LSR Fortessa Flow Cytometer and analyzed with FlowJo software (TreeStar).

### Anti-dsDNA ELISA.

Detection of circulating anti-dsDNA antibodies was carried out as previously described (69) Immunolon 2 plates (Thermo Fisher Scientific) were blocked with methylated-BSA (mBSA) (MilliporeSigma) resuspended in 1x PBS for 30 minutes at 37°C. The plate was then washed twice with 200 μL of 1x PBS, and each well was coated with 50 μg/mL dsDNA for 30 minutes at 37°C. The plate was again washed twice and blocked overnight at 4°C with 3% BSA, 3 mM EDTA (MilliporeSigma), and 0.1% gelatin resuspended in 1x PBS. After blocking, plates were washed twice with 1x PBS. Serum was diluted at 1:250 in 2% BSA, 3 mM EDTA, and 0.05% Tween (Thermo Fisher Scientific) in 1x PBS. This was added to the plate and incubated at room temperature with shaking for 2 hours. The plate was washed twice with 1x PBS + Tween 20 and twice in 1x PBS. IgG-HRP (Promega) was added to each well, diluted at 1:5000 in 1% BSA 0.05% Tween in 1x PBS, and incubated overnight at 4°C with shaking. The plate was then washed twice with 1x PBS + Tween 20. TMB substrate (BD Biosciences) was brought to room temperature, added to the plate, and incubated for 10–15 minutes in the dark. Stop solution was added to the plate, and the plate was read at OD 450 nm immediately.

### Measuring renal IgG deposition.

Kidneys were fixed with formalin and embedded in paraffin before they were sectioned. Each section was incubated in xylene for 8 minutes before being rehydrated in decreasing concentrations of ethanol. Sections were then washed twice with ddH_2_O and placed into 1x PBS. Antigen retrieval was achieved by microwaving sections in 600 mL of ddH_2_O + 5.6 mL of Vector Antigen Retrieval solution concentrate. Sections were then washed with 1x PBS for 5 minutes at room temperature with shaking. Sections were then blocked in 5% normal goat serum in 1x PBS for 1 hour at 37°C. The sections were then stained with biotinylated goat anti-mouse total Ig (Southern Biotech) at a dilution of 1:50 in 5% goat serum in 1x PBS for 1 hour at 37°C. Slides were washed 3 times for 5 minutes with 1x PBS and then incubated with Texas-Red Avidin D (Vector Laboratories) for 1 hour at 37°C. Slides were then washed 3 times with 1x PBS, and VECTASHIELD Antifade Mounting Medium with DAPI (Vector Laboratories) was added before the addition of a coverslip and the imaging of the slide.

### Microscope imaging acquisition.

Sections were imaged at room temperature at 20x on an Olympus BX41 scope fitted with 4x, 10x, 20x, 40x, and 100x oil immersion objectives. This scope was equipped with an Olympus DP71 camera and excited with the X-Cite series 120 Q fluorescence lamp. The images were captured utilizing the manufacturer’s software. Quantification of IgG renal deposition area was performed using ImageJ (NIH) and is expressed as area of IgG staining/area of DAPI staining.

### Islet transplantation.

Islet transplantation was carried out as previously described (27). To render mice chemically diabetic, they were administered streptozotocin via i.p. injection. B6 or SLE123 (H-2b) mice were transplanted with approximately 400 allogeneic C3H islets (H-2k) and assigned to a treatment group that received anti-CD45RB, received anti-CD45RB plus metabolic therapy, or was left untreated. Graft rejection was scored when mice that received grafts demonstrated glucose readings above 250 mg/dL on 2 consecutive days.

### Statistics.

Statistical analysis was performed with GraphPad Prism V5 using 2-tailed Student’s *t* test for comparison of 2 normally distributed conditions. Comparison of multiple groups was conducted using 1- or 2-way ANOVA followed by the Bonferroni posttest. Graft rejection was graphed as a Kaplan-Meier curve and compared by log-rank statistical analysis. Statistical comparisons with *P* ≤ 0.05 were deemed significant. Other statistical analysis utilized was indicated in figure legends.

### Study approval.

The Institutional Animal Care and Use Committee at Vanderbilt University approved all procedures carried out during this study.

## Author contributions

CSW and DJM designed and performed experiments, analyzed data, directed research with collaborators, and wrote and edited the manuscript. BTS, EMH, JPR, KLM, and ASM performed experiments and provided feedback on the manuscript.

## Supplementary Material

Supplemental data

## Version 1 08/17/2021

In-Press Preview

## Version 2 10/08/2021

Electronic publication
