# Supplementary material for: Metabolic preconditioning in CD4+ T cells restores inducible immune tolerance in lupus-prone mice
Source: JCI Insight. 2021 Oct 8;6(19):e143245. doi: 10.1172/jci.insight.143245 (PMC8525586; doi:10.1172/jci.insight.143245)
Supplement: Supplemental data [file jciinsight-6-143245-s008.pdf]

Supplemental 1

A

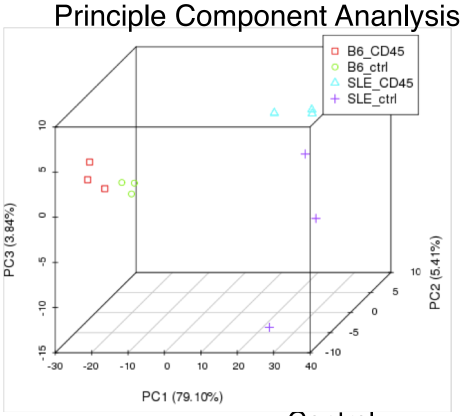

B

Downregulated Genes associated with KEGG term "metabolic pathways"

Coasy	Mccc2	Dtymk	Pts	B3galt6
Sgsh	Adsl	Trdmt1	Stt3b	Ndst1
Mthfd2	Ehhadh	Mccc1	Pdxk	Ppox
Ggt5	Gart	Ahcyl1	Tk2	Ppcdc
St3gal1	Synj2	Pigo	Acaa2	ND1
Pigl	Man2a1	Ctps	Galnt6	ND2
Dguok	Gpam	H6pd	Galnt11	COX1
Adss	Prps2	Coq2	Man2a2	ND4
Adi1	Pgm2	St8sia1	Pigk	ND5
Pfas	Inpp1	Idh2	Plcb2	ND6
Coq6	Gls	Pik3c2a	Hibch	CYTB
Aldh6a1	Mgat4a	Ears2	Pigm	Aoc2

C

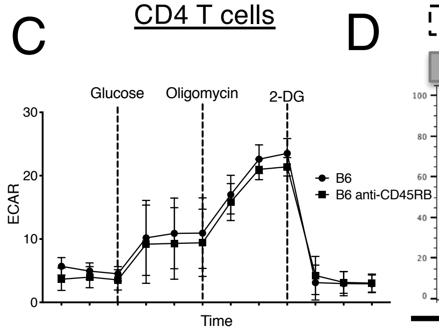

D

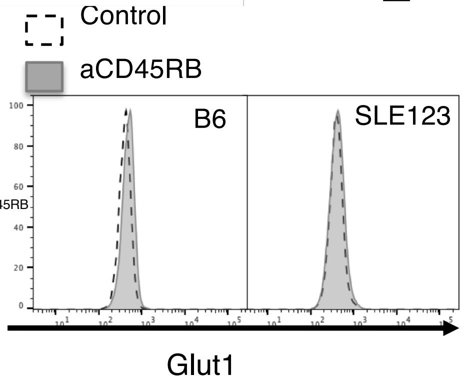

E

Compound	p-value	Regulated	FC
4-Methylhistamine	0	up	16.00
6,8-Dihydroxypurine	0	up	16.00
6,8-Dihydroxypurine	0	up	16.00
Nitrilacarb	0	up	16.00
Glyodin	0	up	16.00
docusate	0	up	16.00
GalCer(d18:0/16:0)	3.81E-04	up	2.60
GlcCer(d18:2/22:0)	0.00429552	up	2.54
Galbeta-Cer(d18:1/22:0)	0.03171751	up	2.38
PGH2-EA	2.73E-04	up	2.33
PS(P-20:0/19:1(9Z))	7.77E-05	up	2.30
PE(24:0/18:2(9Z,12Z))	1.85E-05	up	2.26
CerP(d18:1/24:0) Esi-1.3639991	0.00750449	up	2.23
Galbeta-Cer(d18:1/16:0)	3.81E-04	up	2.22
PS(20:3(8Z,11Z,14Z)/18:0)	5.72E-04	up	2.21
O6-Methyl-2'-deoxyguanosine	2.73E-04	up	2.19
O6-Methyl-2'-deoxyguanosine	0.02108133	up	2.17
PE(O-20:0/22:0)	4.50E-05	up	2.13
Galbeta-Cer(d18:1/16:0)	1.07E-04	up	2.11
PS(O-16:0/20:0)	1.85E-05	up	2.04
PS(O-20:0/19:1(9Z))	2.30E-05	up	2.03
PE(24:0/18:1(9Z))	0.00864221	up	2.01
PS(O-18:0/21:0) Esi-1.3450006	0.03045165	up	2.00
Fexofenadine	0.00633	down	-2.04
Fexofenadine	0.00633477	down	-2.06
Guanosine	0	down	-2.12
4-Hydrocinnamoyl-2,2,5-trimethyl-4-cyclopentene-1,3-dione	0.01719775	down	-2.15
Cimicifugoside	0.02636899	down	-2.36
Chryso-obtusin glucoside	3.85E-04	down	-2.73
1-Naphthalenesulfonic acid	1.85E-05	down	-2.89
Inosine	0	down	-16.00
Guanosine	0	down	-16.00

F

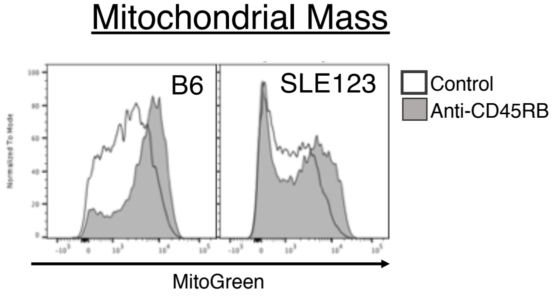

G

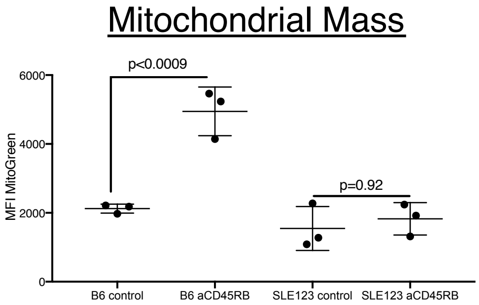

Supplemental Figure 1. Metabolic parameters are altered in B6 CD4+ T cells treated with aCD45RB but resisted by SLE123 mice. A) PCA analysis of RNA sequencing data. **B)**This is a complete list of the genes associated with the downregulated "Metabolic Pathways" KEGG term in B6 CD4+ T cells treated wit aCD45RB. **C)** Seahorse analysis of purified CD4+ T cells from B6 mice treated with aCD45RB or left untreated revealed no difference in glycolysis among these cells. **D)** Analysis of Glut1 expression in B6 and SLE123 CD4+ T cells revealed no difference in the expression of Glut1 between aCD4RB treated and untreated groups. This analysis was carried out on fixed and permeabilized CD4+ T cells as this antibody detects an intracellular portion of Glut1. **E)** Untargeted metabolomics revealed an increase in 6,8-dihydroxypurine, an indicator of DNA damage. This correlates well with the downregulation of genes associated with the Pentose Phosphate Pathway and Purine and Pyrimidine Biosynthesis, responsible for maintaining the nucleotide pool in CD4+ T cells. **F)** CD4+ T cells from B6 mice treated with aCD45RB demonstrated increased mitochondrial mass when compared to B6 untreated. SLE123 CD4+ T cells resisted these changes. Quantified in **G**.

Supplemental 2

KEGG Analysis

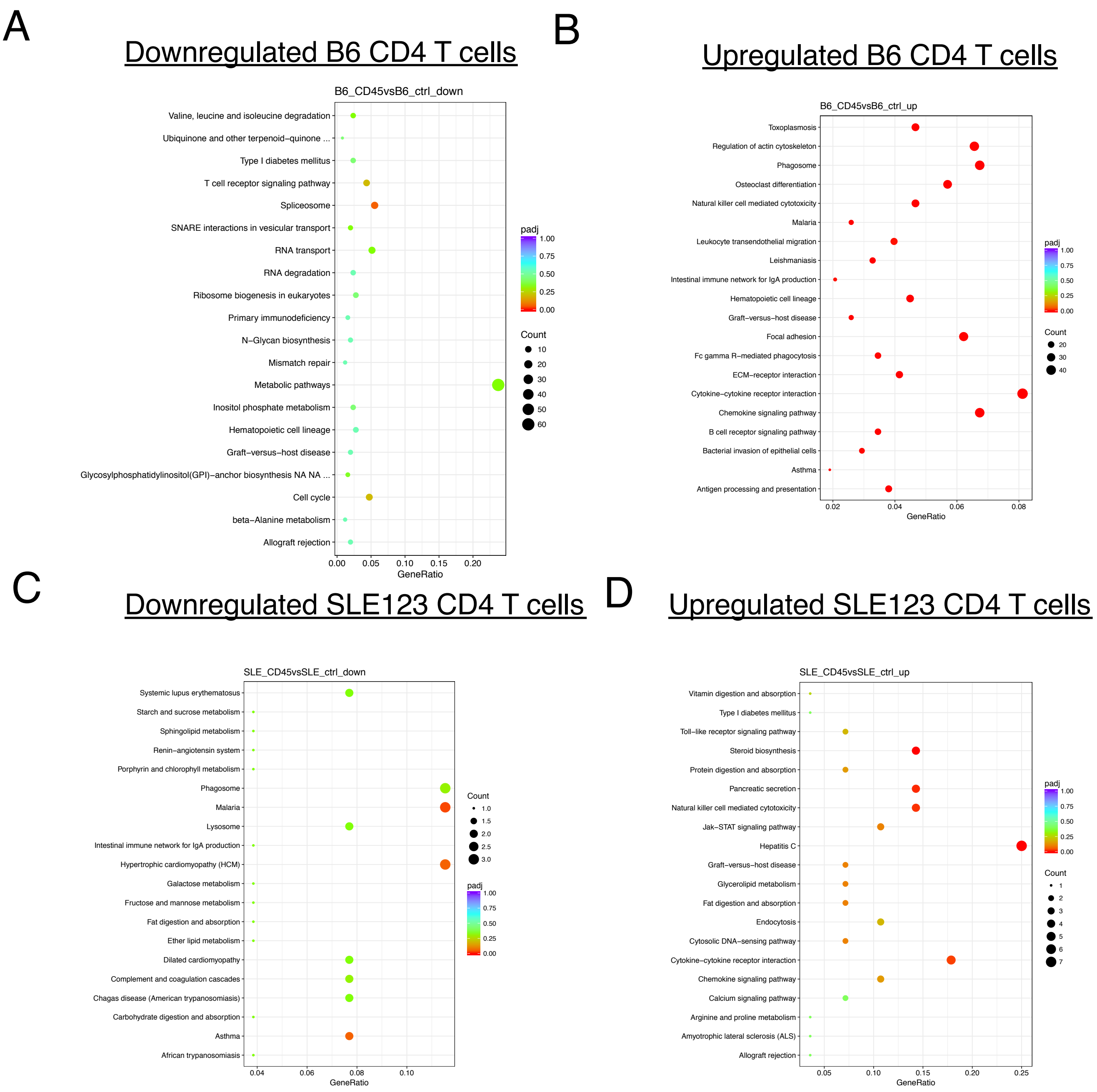

Supplemental Figure 2. KEGG analysis of genes identified in RNA sequencing of purified CD4 T cells from SLE123 and B6 mice treated with anti-CD45RB. A-D. KEGG pathways identified by analysis of RNA sequencing data. The size of the dot for each term indicates the count of genes found, while the x-axis indicates its gene ratio. The color represents the adjusted p-value.

Supplemental 3

GO Analysis

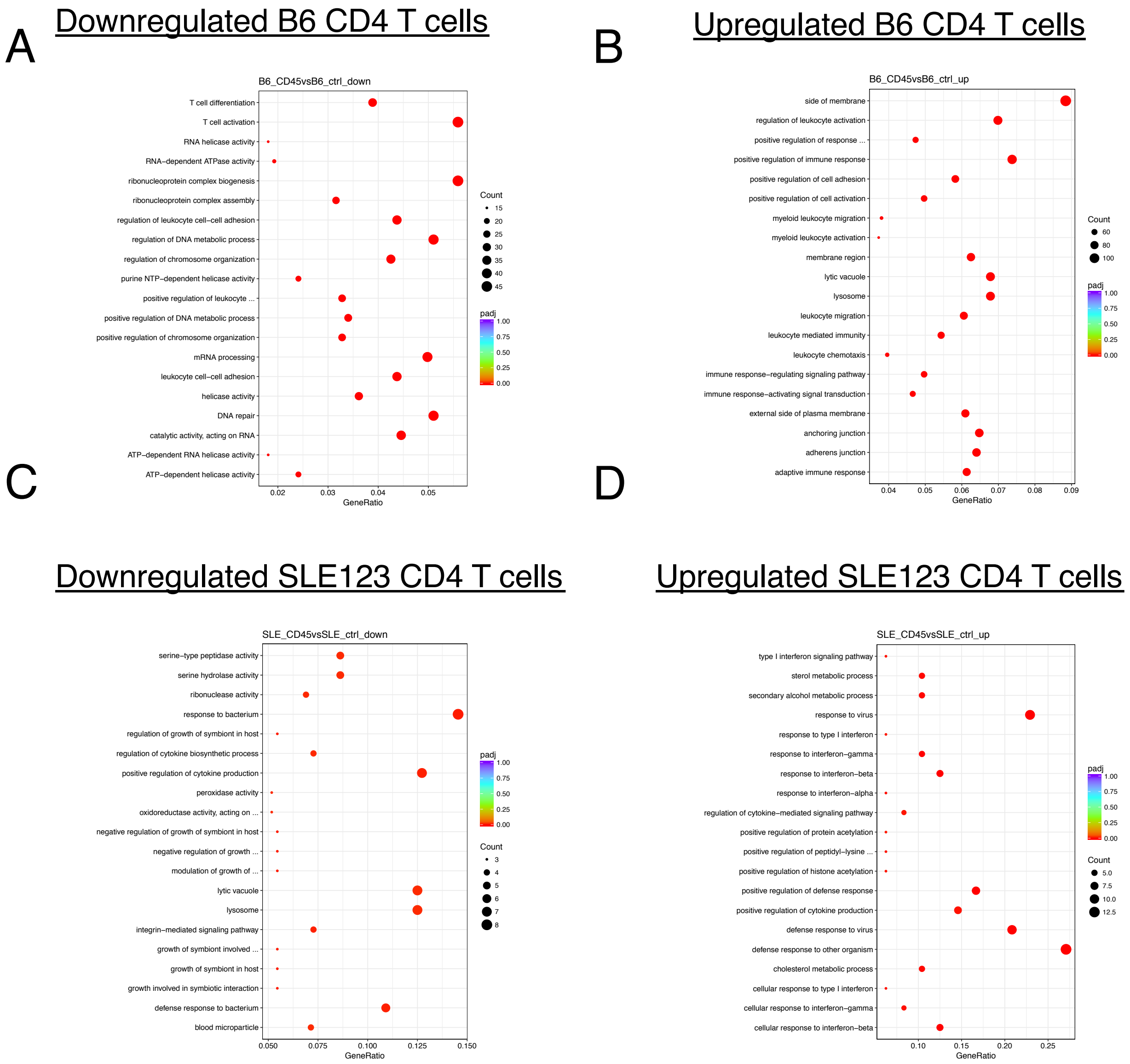

Supplemental Figure 3. GO analysis of genes identified in RNA sequencing of purified CD4 T cells from SLE123 and B6 mice treated with anti-CD45RB.A-D. GO pathways identified by analysis of RNA sequencing data. The size of the dot for each term indicates the count of genes found, while the x-axis indicates its gene ratio. The color represents the adjusted p-value.

Supplemental 4

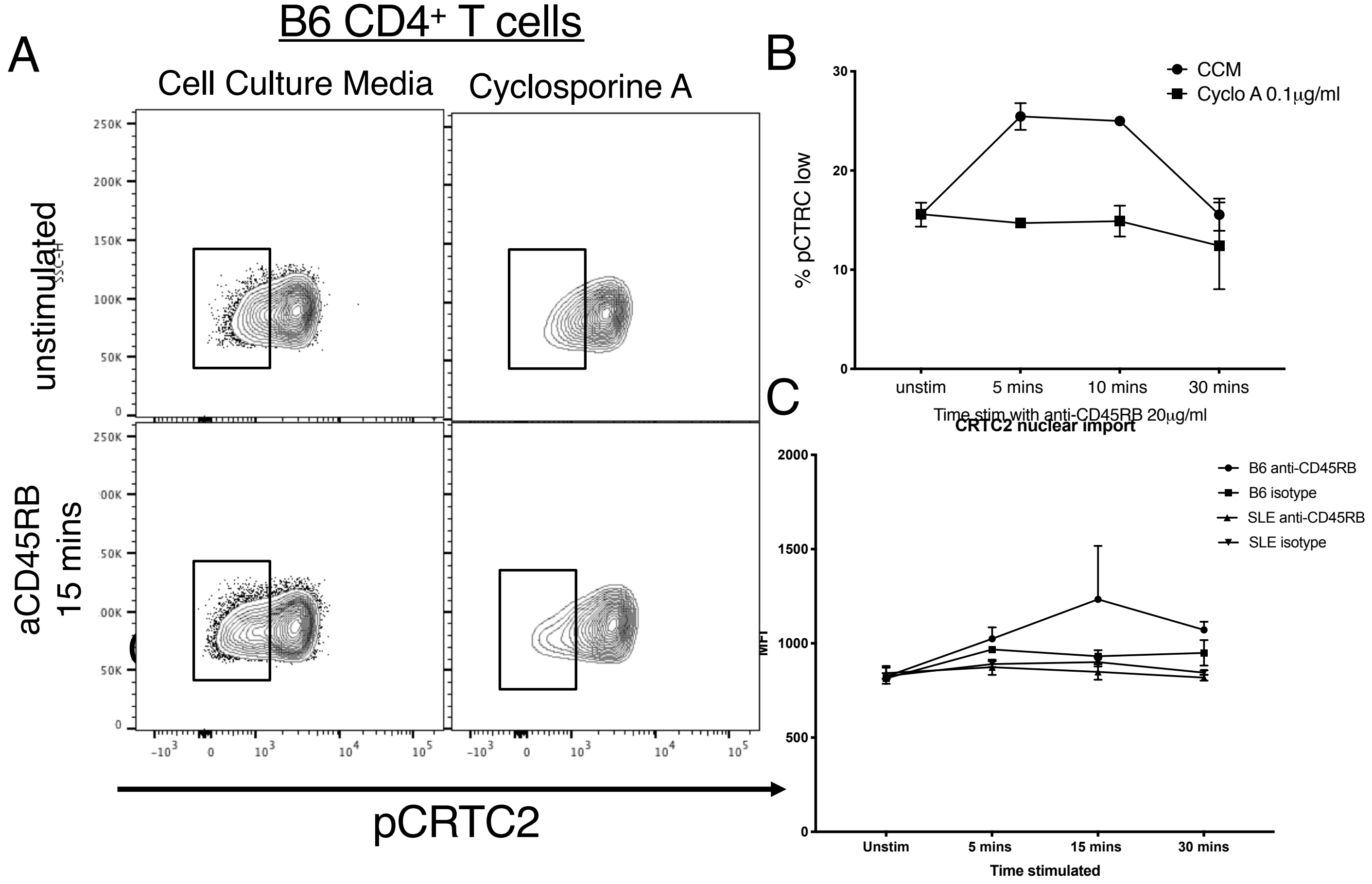

Supplemental Figure 4. aCD45RB induces dephosphorylation and nuclear import of CRTC2. A) CD4+ T cells from B6 mice were incubated with aCD45RB in culture media or in the presence of calcineurin inhibitor cyclosporine A. B6 CD4+ T cells demonstrated a decrease in phosphorylation when incubated with aCD45RB for 15mins (bottom left panel) as compared to unstimulated (top left panel), indicating aCD45RB drove dephosphorylation of CRTC2. Incubation of CD4+ T cells with cyclosporine A before aCD45RB prevented dephosphorylation (left panel). **B)** This graph shows the percentage of cells that had reduced phosphorylation of CRTC2 after aCD45RB. Dephosphorylation peaks and then declines by 30 mins. Those cells preincubated with cyclosporine A demonstrated little CRTC2 dephosphorylation. **C)** CRTC2 nuclear import was only observed in B6 CD4+ T cells treated with aCD45RB. Shown here is the kinetics of nuclear import. CRTC2 nuclear import peaks around 15 mins, following peak dephosphorylation of CRTC2. B6 isotype control treated and SLE123 CD4+ T cells, regardless of treatment, demonstrated little nuclear import of CRTC2 as measured by isolated nuclei via flow cytometry. n=3 9-12 week old female mice per experiment.

Supplemental 5

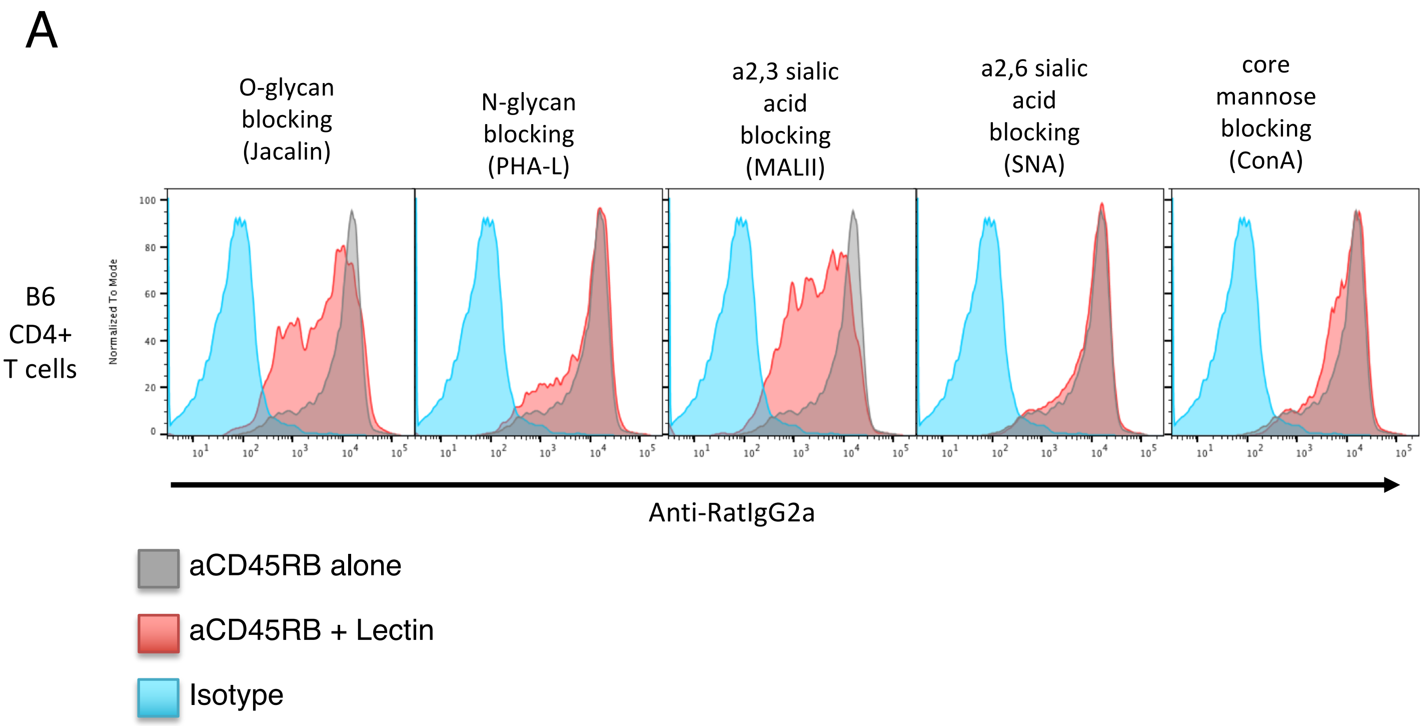

Supplemental Figure 5. Lectins that block O-glycosylation (Jacalin) and a2,3-linked sialic acid moieties (MALII) prevent aCD45RB binding. A) To determine what glycosylation patterns are important for aCD45RB binding to CD4+ T cells, we incubated splenocytes with the lectins indicated above. aCD45RB or isotype followed by an anti-RatIgG2a conjugated to PE was added for detection of binding. Lectins that target O-glycosylation (Jacalin) and a2,3-linked sialic acids (MALII) inhibit aCD45RB binding the most. Representative data from 3 experimental repeats. Carried out on 9-12 week old female B6 mice.
